# Multiple Evanescent White Dot Syndrome Presenting After Initiation of Empagliflozin, a Sodium-Glucose Cotransporter-2 Inhibitor

**DOI:** 10.7759/cureus.13853

**Published:** 2021-03-12

**Authors:** Heidi Boutros, Chinwenwa Okeagu, Aman Sharma, Saad Shaikh

**Affiliations:** 1 Ophthalmology, University of Central Florida, Orlando, USA; 2 Ophthalmology, National Eye Institute/National Institutes of Health, Bethesda, USA; 3 Ophthalmology, University of Central Florida College of Medicine, Orlando, USA; 4 Ophthalmology, Johns Hopkins, Baltimore, USA; 5 Ophthalmology, Orlando Veterans Affairs Medical Center, Orlando, USA; 6 Ophthalmology, University of Texas Medical Branch Galveston, Galveston, USA; 7 Ophthalmology, Howard University College of Medicine, Washington, DC, USA

**Keywords:** diabetes mellitus, empagliflozin, mewds, multiple evanescent white dot syndrome, sodium-glucose co-transporter 2 inhibitor, sglt2 inhibitor

## Abstract

Multiple evanescent white dot syndrome (MEWDS) is an inflammatory condition of the retina that typically presents unilaterally with multiple gray-white spots in the outer retina or retinal pigmented epithelium and granular changes within the fovea. We report a case of new-onset MEWDS in a patient closely after the initiation of empagliflozin, a sodium-glucose cotransporter inhibitor medicine for his type II diabetes mellitus.

## Introduction

Multiple evanescent white dot syndrome (MEWDS) is a self-limited disease that is typically seen in young females. Clinical findings include multifocal white lesions located in the posterior pole of the retina that demonstrate punctate hyperfluorescence with late staining on angiography. Here, to our knowledge, we report the first case of MEWDS following treatment with sodium-glucose cotransporter-2 (SGLT2) inhibitor therapy (empagliflozin).

## Case presentation

A 52-year-old male with diabetes mellitus was referred to our service for a one-week history of vision loss in his right eye. The patient had recently begun an SGLT2 inhibitor, empagliflozin (Jardiance®), 12 mg PO qam, 10 days prior to onset of symptoms. He had a history of myopia, lattice degeneration, and narrow and open-angle glaucoma. His medical history was otherwise non-contributory. No recent history of viral prodrome symptoms or exposure to recent viral infections was reported.

**Figure 1:**
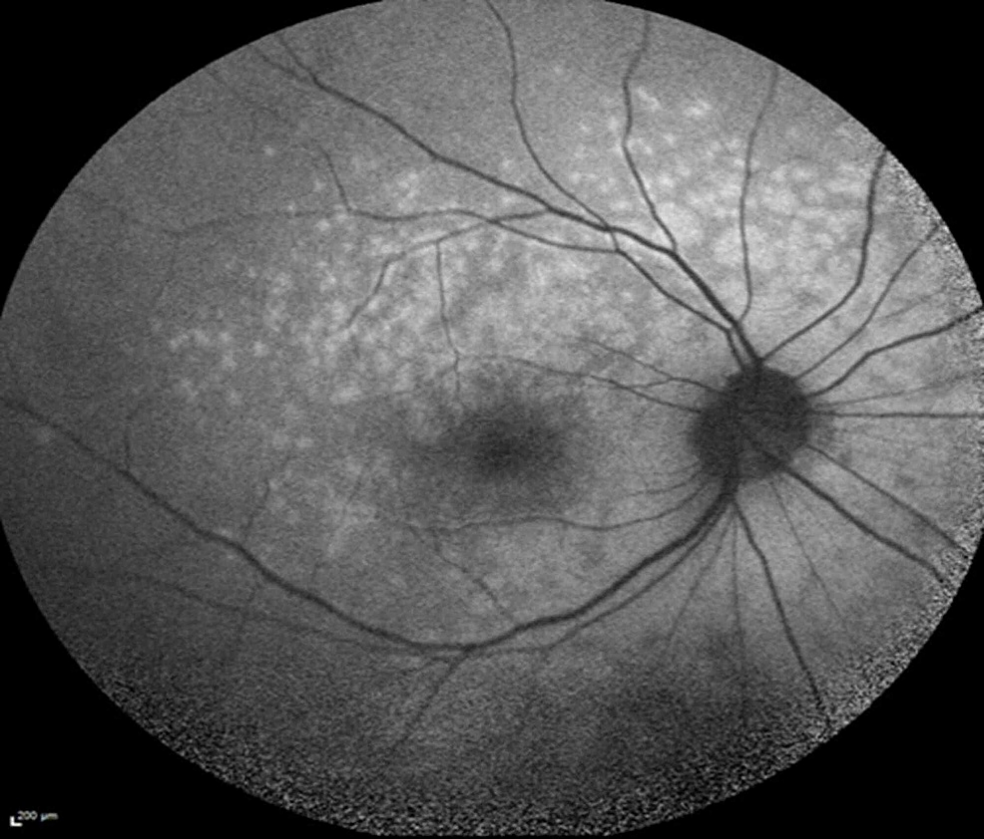
Fundus autofluorescence on presentation revealed numerous hyperautoflorescent dots in the right eye.

**Figure 2:**
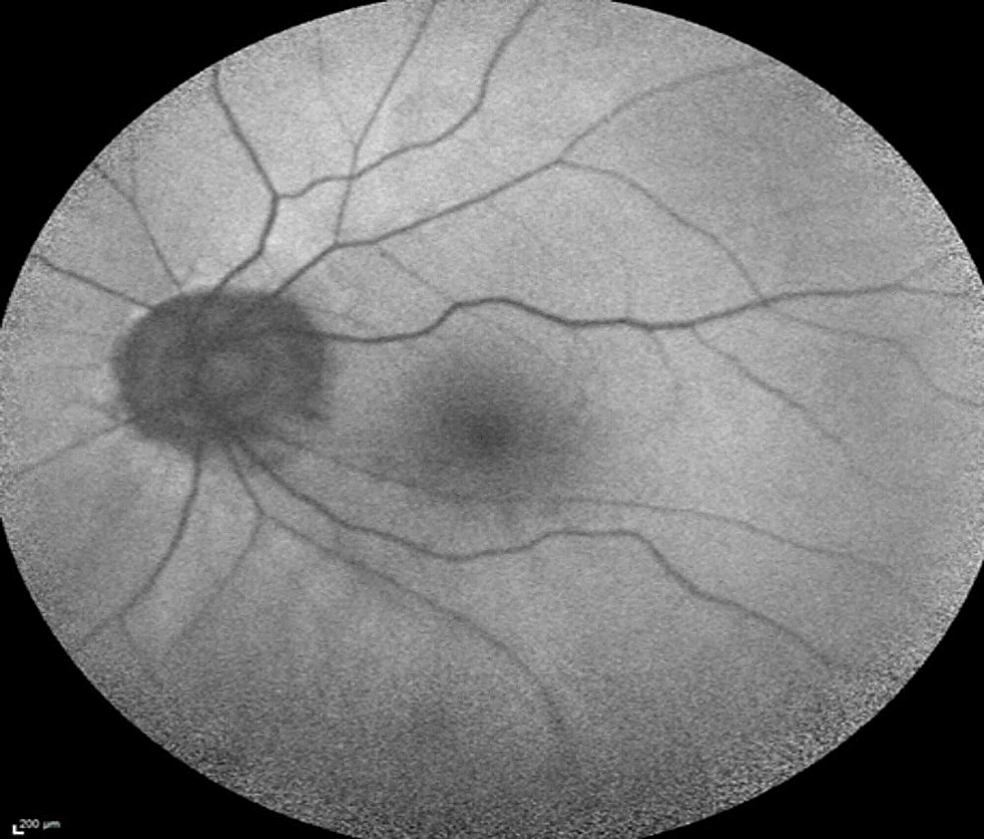
Normal fundus autofluorescence image of the left eye on presentation.

On presentation, his best-corrected visual acuity was 20/20-2 OD and 20/40-2 OS. Anterior segment examination was unremarkable with patent peripheral iridectomies OU. Funduscopic examination demonstrated tilted optic nerves with sharp margins in both eyes and a previously documented long-standing disc hemorrhage in the left eye. Multiple gray-white subretinal lesions of varying size were noted in the posterior pole of the right eye. No lesions were noted in the left eye. Lattice degeneration was observed in both eyes peripherally. The vitreous was clear without cell or debris in either eye. Fundus autofluorescence revealed numerous hyperautoflorescent dots in the right eye (Figure 1) and the normal left eye (Figure 2).

**Figure 3:**
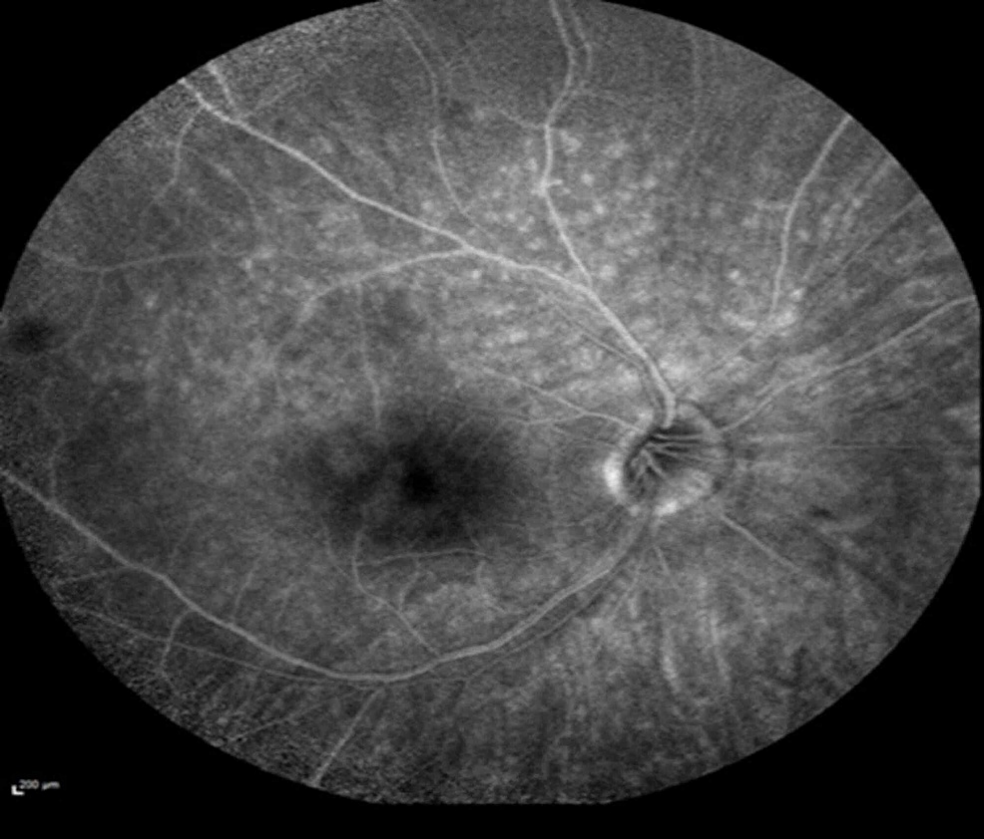
Fluorescein angiography on presentation showed punctate areas of early hyperfluorescence in the right eye.

**Figure 4:**
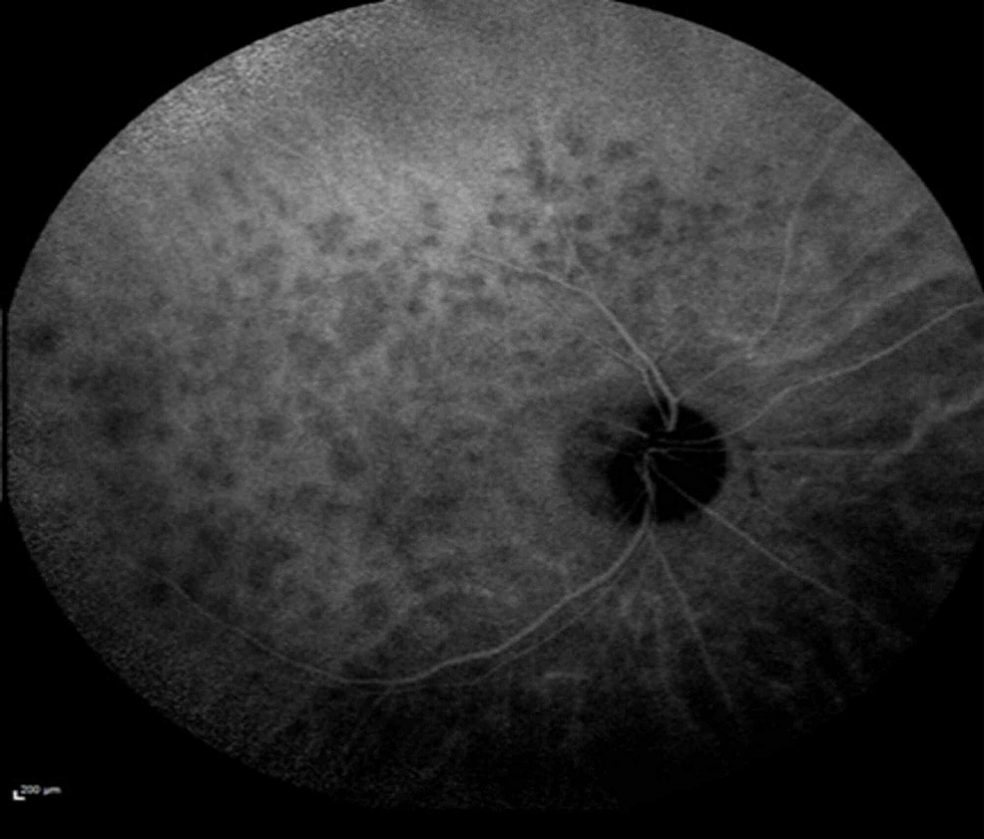
Indocyanine green angiography on presentation showed numerous hypocyanscent lesions in the right eye.

Fluorescein angiography demonstrated punctate areas of early hyperfluorescence in the right eye (Figure 3), and indocyanine green angiography demonstrated hypofluorescence of the same lesions (Figure 4).

**Figure 5:**
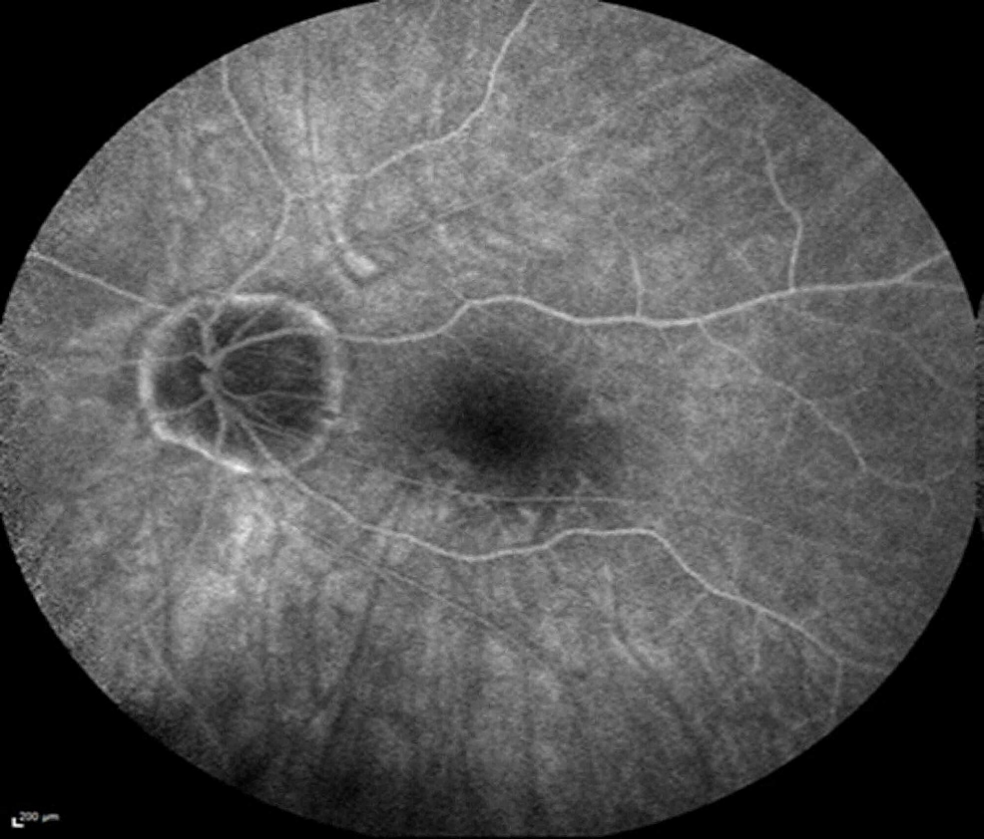
Normal fluorescein angiography of the left eye on presentation.

**Figure 6:**
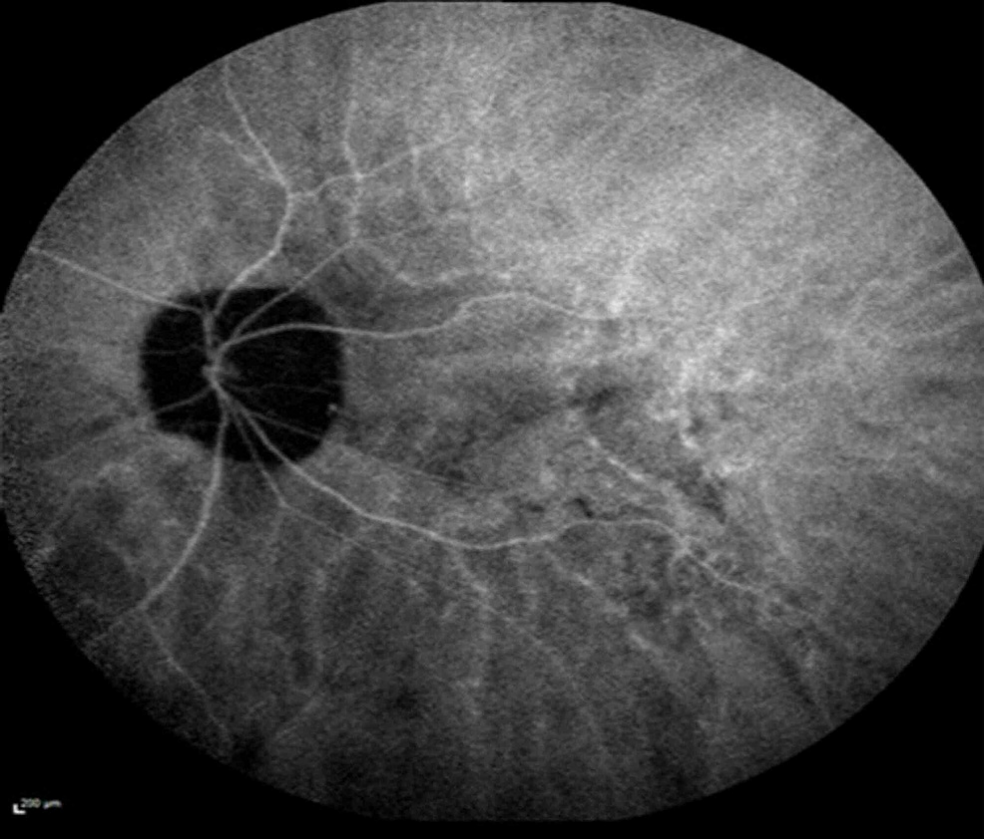
Normal indocyanine green angiography of the left eye on presentation.

The left eye was unremarkable (Figures 5, 6).

**Figure 7:**
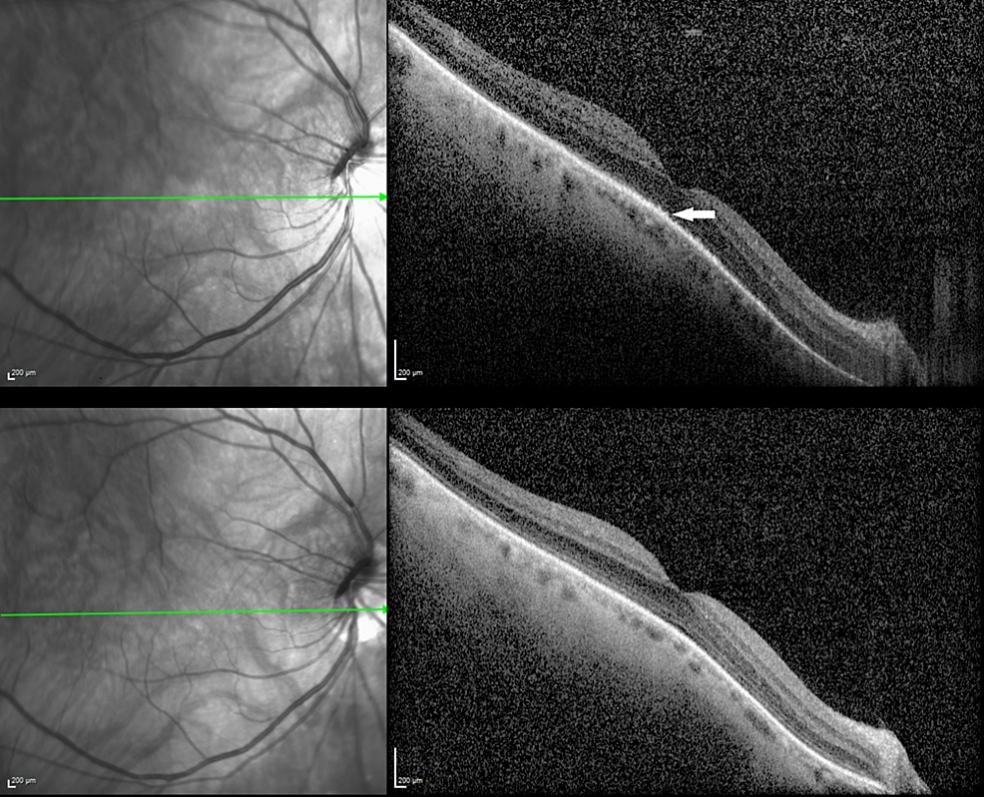
OCT showed disruption of the ellipsoid zone and outer segment abnormalities (arrow) in the right eye on presentation (top). Normal OCT on final follow-up with restoration of outer retinal anatomy (bottom). OCT, optical coherence tomography

Laboratory studies including complete blood count with differential, QuantiFERON, and syphilis testing were negative, as was magnetic resonance imaging of the brain and orbits. The medication was discontinued and he was started on empiric antiviral medication (valganciclovir 900 mg po bid for 10 days) and steroids (prednisone 100 mg po daily with taper over three weeks). Ancillary imaging was repeated at six weeks and showed resolution of the white lesions, and best-corrected visual acuity was 20/25 OU. The optical coherence tomography images that had demonstrated disruption of the ellipsoid zone and outer segment abnormalities on initial presentation returned to normal (Figure 7). He did not resume empagiflazolin therapy.

## Discussion

SGLT2 inhibitors, also called gliflozins, are a relatively new class of drugs approved since 2012 for the treatment of diabetes mellitus. Inhibition of the SGLT2 transporter in the proximal convoluted tubule of the kidney prevents the reabsorption of glucose from urine, resulting in reduced serum glucose levels in an insulin-independent manner. Our patient presented with MEWDS soon after initiation of SGLT2 inhibitor therapy. To date, no previous cases of MEWDS or other white dot syndromes have been reported after SGLT2 inhibitor therapy. The etiology of MEDWS is not completely understood although an auto-immune mechanism is implicated in its pathogenesis. Previous case reports note the development of MEWDS after hepatitis A and human papillomavirus vaccinations [1,2]. Initially, SGLT2 receptors were thought to be located only in the kidney, but they have subsequently been discovered in retinal pericytes, mesangial cells, and endothelial cells [3]. They have been found to be upregulated in the retina in patients with diabetes where they also serve a critical role in retinal glucose uptake, transport, and subsequent diabetes-induced retinal vascular remodeling and damage [3,4]. Recent case reports have implicated empagliflozin in a variety of drug-induced myopathies [5,6]. We hypothesize that our patient developed retinal involvement through direct toxicity from binding of the medication to retinal cells and a subsequent inflammatory and/or immune-mediated response.

## Conclusions

In summary, we report the association of MEWDS with SGLT2 inhibitor therapy. In this patient, the course was self-limited on cessation of the medication and steroid therapy. Further studies may be necessary to determine the incidence of this condition in the wider diabetic patient population on this class of medications.
